# The role of the classical renin–angiotensin system and angiotensin-converting enzyme 2/Ang(1–7)/Mas axis in pulmonary fibrosis

**DOI:** 10.3389/fmed.2025.1615991

**Published:** 2025-07-29

**Authors:** Changhui Lang, Bo Huang, Yan Chen, Zhixu He

**Affiliations:** ^1^Department of Pediatrics, Affiliated Hospital of Zunyi Medical University, Zunyi, Guizhou, China; ^2^Guizhou Children's Hospital, Zunyi, Guizhou, China

**Keywords:** pulmonary fibrosis, angiotensin-converting enzyme 2, angiotensin 1–7, angiotensin II, renin-angiotensin system

## Abstract

Pulmonary fibrosis (PF), a progressive and fatal disease, is characterized by fibroblast proliferation, excessive extracellular matrix deposition, and collagen synthesis. These pathological changes lead to impaired lung structure and function, ultimately resulting in respiratory failure. Emerging basic and clinical evidence highlight the renin–angiotensin system (RAS) as a critical contributor to PF onset and progression. Angiotensin (Ang) II, a key RAS component, mediates various biological effects through its receptors, Ang II receptor type 1 (AT_1_R) and Ang II receptor type 2 (AT_2_R). Ang II promotes vasoconstriction, inflammation, and fibrosis *via* AT_1_R, while it shows contrasting effects through AT_2_R. Angiotensin-converting enzyme 2 (ACE2) plays a significant role in RAS by converting Ang II into Ang (1–7), which in turn interacts with Mas receptor and Mas-associated G-protein-coupled receptor D to exert anti-inflammatory, anti-apoptotic, and anti-fibrotic effects. The RAS also influences autophagy, oxidative stress, and inflammation in the progression of PF. This review provides an updated overview of the roles of the classical and non-classical RAS pathways in PF.

## 1 Introduction

Pulmonary fibrosis (PF) is a rapidly progressive and fatal condition with high morbidity and mortality, often secondary to acute lung injury (ALI) and acute respiratory distress syndrome (ARDS). PF is characterized by repetitive epithelial injury, epithelial–mesenchymal transition (EMT), endothelial–mesenchymal transition, cell senescence, fibroblast activation, proliferation, extensive extracellular matrix (ECM) accumulation, lung architectural distortion, and pulmonary dysfunction. Its etiology remains unknown. PF leads to a gradual decline in lung function, resulting in end-stage respiratory failure (1, 2). In the context of the complexity of PF pathogenesis, current treatments, such as pirfenidone and nintedanib, primarily aim to slow fibrosis progression. However, an optimal therapeutic strategy for PF has yet to be established (3–5). Therefore, identifying novel therapeutic targets for PF remains critically important.

The renin–angiotensin system (RAS) is widely recognized for its essential role in regulating blood pressure, electrolyte balance, and blood volume. Components of RAS are identified in various organs, including the heart, blood vessels, lungs, and kidneys (6). It comprises two subsystems: the classical RAS and the non-classical or alternative RAS (7). The classical RAS primarily includes angiotensin-converting enzyme (ACE), angiotensin (Ang) II, and the Ang II receptor type 1 (AT_1_R). In this system, renin converts angiotensinogen (AGT) into Ang I, a substrate for the ubiquitously expressed ACE, particularly in lung tissue. ACE further processes Ang I into Ang II, the key effector of the classical RAS, which exerts various physiological effects by binding to specific receptors, AT_1_R and AT_2_R (8). Ang II promotes vasoconstriction, pro-inflammatory, pro-apoptotic, and pro-fibrotic activities, as well as sodium balance regulation, primarily through AT_1_R. However, Ang II has been shown to elicit opposing effects when interacting with AT_2_R (9). Ang II is degraded by ACE2, a key regulator that counteracts the effects of the classical RAS (10). Ang II can also be hydrolyzed by aminopeptidase A (APA) into Ang III and then converted to Ang IV by aminopeptidase N (APN) (11). Ang II activates a range of intracellular protein kinases, including receptor tyrosine kinases such as epidermal growth factor receptor (EGFR) and platelet-derived growth factor receptor (PDGFR), as well as non-receptor tyrosine kinases such as Src, which is upregulated in PF and accelerates the release of transforming growth factor-β1 (TGF-β1). Moreover, Ang II stimulates serine/threonine kinases such as mitogen-activated protein kinase (MAPK), Akt/protein kinase B, and various protein kinase C isoforms (12).

The alternative RAS, comprising ACE2, Ang (1–7), and the Mas receptor (MasR), plays vasodilatory, anti-inflammatory, and anti-fibrotic roles in respiratory diseases such as ARDS (13). In the non-classical RAS, ACE2 cleaves Ang I to produce the Ang (1–9) peptide, which counteracts the ACE arm. Ang (1–9) can later be converted into Ang (1–7) by ACE or neprilysin (NEP) (14). NEP (15), a membrane metalloendopeptidase (MME), directly processes Ang I into Ang (1–7), improving its protective effects, particularly in the lung, especially in the presence of ACE inhibitors (16). NEP also hydrolyzes endothelin-1 (ET-1), a known bronchoconstrictor and vasoconstrictor in the airways, mitigating inflammatory responses and preventing the fibrotic cascade in the lung (17). The increase in plasma ET-1 levels is linked to Ang II release. ET-1 contributes to pulmonary vascular remodeling, potentially leading to pulmonary arterial hypertension (PAH) secondary to bleomycin (BLM)-induced PF (14, 18). ET-1 also stimulates the release of TGF-β1 following severe acute respiratory syndrome coronavirus 2 (SARS-CoV-2) infection. This process drives endothelial dysfunction that can result in vascular constriction and increased vascular permeability (19, 20). Ang (1–7), which is hydrolyzed by ACE into Ang (1–5), was initially considered biologically inactive (14). However, recent evidence suggests that Ang (1–5) promotes NO release by activation of eNOS via interaction with the AT_2_R (21).

**Figure 1:**
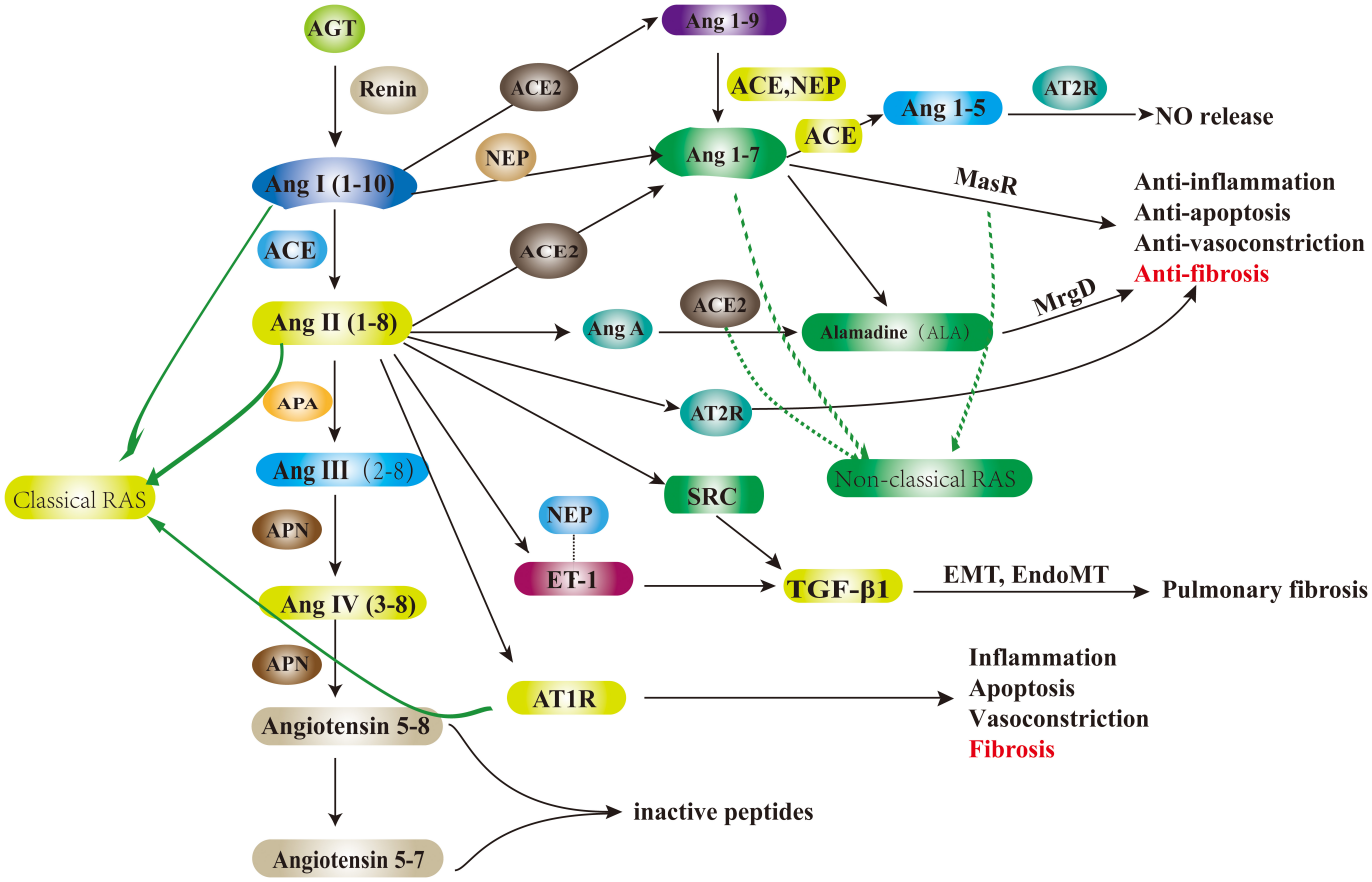
Schematic diagram of the local renin–angiotensin system and ACE2/Ang (1–7)/Mas axis in pulmonary fibrosis. AGT, angiotensinogen; Ang I, angiotensin I; Ang II, angiotensin II; Ang III, angiotensin III; Ang IV, angiotensin IV; ACE2, angiotensin-converting enzyme2; NEP, neprilysin/neutral endopeptidase; ACE, angiotensin-converting enzyme; ET-1, endothelin-1; AT2R, angiotensin II type 2 receptor; AT1R, angiotensin II type 1 receptor; APA, aminopeptidase A; APN, aminopeptidase N; Ang A, angiotensin A; TGF-β1, transforming growth factor-β1; EMT, epithelial–mesenchymal transition; NO, nitric oxide; EndoMT, endothelial–mesenchymal transition; MasR, Mas receptor; MrgD, Mas-related G-protein-coupled receptor D.

The non-classical RAS also facilitates the conversion of Ang II into the vasodilator Ang (1–7) *via* ACE2, thereby counteracting the effects of Ang II. Furthermore, ACE2 degrades Ang A (an aspartate-to-alanine homolog of Ang II) into another vasodilator, alamandine (ALA) (22). ALA interacts with the Mas-related G-protein-coupled receptor D (MrgD), playing a protective role in opposing the classical RAS and mitigating fibrosis (23) (Figure 1).

Under normal physiological conditions, the classical and non-classical RAS maintain a delicate balance. Emerging evidence indicates that the dysregulation of the RAS is associated with the progression of various diseases, particularly cardiovascular diseases and PAH (24), chronic obstructive pulmonary disease (COPD) (8), and ALI (25). The RAS also plays a critical role in regulating various cellular processes, including inflammation, proliferation, and apoptosis. It is also implicated in endothelial dysfunction and vascular remodeling in animal models of PAH (26, 27). ACE2, a key component of the non-classical RAS, is closely associated with PAH. Reduced ACE2 activity is closely linked to the development of PAH, while its upregulation has been shown to improve pulmonary homeostasis, reduce oxidative stress, and suppress inflammatory mediators (28). The ACE2 activator diminazene aceturate (DIZE) alleviates monocrotaline-induced PAH and restores the imbalance caused by monocrotaline (29). Increasing evidence supports the link between dysregulated RAS and the development and progression of PF (30, 31). Previous studies have shown that local RAS activation contributes to PF development induced by hyperoxia in neonatal rats (32). Moreover, ACE inhibitors and non-selective Ang II receptor antagonists, such as saralasin, effectively block experimental lung fibrosis in animal models (9).

Further research is needed to fully understand the role of the classical RAS and the ACE2/Ang (1–7)/Mas network in PF. The ACE2/Ang (1–7)/Mas network may serve as a potential therapeutic target for PF. This review offers a comprehensive overview of the relationship between the classical RAS and the ACE2/Ang (1–7)/Mas network in PF.

## 2 The roles of the ACE/Ang II/AT_1_R pathway in PF

The ACE/Ang II/AT_1_R network is a key regulator in the development of PF (30, 31). Elevated renin levels have been observed in the lungs and fibroblasts of patients with PF, correlating with increased expression of TGF-β1. This cytokine is pivotal in driving fibrosis by promoting fibroblast activation and ECM deposition, resulting in tissue scarring and compromised organ function. Furthermore, renin suppresses the expression of matrix metalloproteinase-1 (MMP-1), an enzyme involved in ECM degradation. Knocking down renin results in a significant decrease in TGF-β1 levels (33).

Ang II is the primary effector in the classical RAS system. Ang II can stimulate the formation of fibrosis *via* AT_1_R in various tissues, including the heart, kidney, and lungs (34). Elevated levels of Ang II and AT_1_R have been observed in a PF rat model induced by BLM, and inhibiting Ang II alleviates structural damage to lung tissue (35). Treatment with AT_1_R antagonists has been shown to reduce the expression of alpha-smooth muscle actin (α-SMA) in PF induced by hyperoxia in neonatal rats (36). An increase in Ang II leads to the accumulation of collagen in the lungs (37). Furthermore, the rise in TGF-β1 and collagen deposition caused by Ang II was blocked by AT_1_R-selective antagonists such as L158809 or losartan (9). Following BLM exposure, the severity of lung fibrosis and the hydroxyproline levels were significantly reduced by the AT_1_R antagonist olmesartan medoxomil (38).

As previously mentioned, Ang II has antifibrotic effects when binding to AT_2_R (9). The AT_2_R agonist compound 21 prevented the development of lung fibrosis induced by BLM at day 0 or halted its progression at day 3 (39), suggesting that Ang II plays distinct roles depending on which receptor it binds to. Ang II plays a significant role in signaling pathways critical to fibrosis pathogenesis. The primary pathway mediated by BLM in PF is the activation of the small mothers against decapentaplegic homologs (Smad)/TGF-β signaling cascade (40). Elevated levels of collagen I, AT_1_R, TGF-β1, and phosphorylated Smad2/3 (p-Smad2/3) have been detected in lung fibroblasts stimulated with macrophage-derived exosomes following Ang II exposure (41). Lung fibroblasts isolated from patients with PF produce Ang II, AGT, and α-SMA, which colocalize within myofibroblast foci (42). Inhibition of Ang II signaling reduces myofibroblast differentiation and ECM deposition in silicotic fibrosis models (43). Furthermore, targeting both the Ang II/AT_1_R axis and the TGF-β/Smad signaling pathway alleviates BLM-induced lung fibrosis (40).

The expression of AT_1_R is also upregulated in lung tissues affected by silicotic fibrosis (43). Elevated ACE, Ang II, and AT_1_R levels have been linked to right ventricular hypertrophy and hypoxia-induced fibrosis (44). The increased AT_1_R and reduced MasR have been observed in patients with PF. AT_1_R expression is inversely correlated with pulmonary function (45). Furthermore, the co-expression of ACE and AT_1_R in alveolar epithelial cells was significantly elevated in PF following mechanical ventilation (46). AT_1_R antagonists, such as losartan, or genetic disruption of the AT_1_R gene, reduce hydroxyproline accumulation and caspase-3 activity both *in vitro* and *in vivo*, including in models of lung fibrosis (47).

AT_1_R antagonists, such as losartan, have been shown to significantly improve lung function in patients with PF over 1 year (48). However, these findings require validation through larger, controlled clinical studies. Inhibition of Ang II synthesis using ACE inhibitors, including captopril and enalapril, has also been reported to reduce hydroxyproline content and TGF-β1 levels in animal models of PF (9, 49). Clinically, treatment with ACE inhibitors (e.g., lisinopril and ramipril) or AT_1_R antagonists (e.g., valsartan and losartan) has been associated with decreased mortality risk and a slower rate of FVC decline in patients with PF, suggesting a potential disease-modifying effect in PF compared to patients not receiving ACE inhibitor or AT_1_R therapy (50). However, the interpretation of these findings is constrained by the retrospective nature of the exploratory analyses, which revealed associations between ACE inhibitor or Ang receptor blocker (ARB) use and clinical outcomes without establishing causality. These analyses are limited to patients receiving placebo treatment. Therefore, further prospective studies are needed to clarify the therapeutic impact of ACE inhibitors or AT_1_R antagonists, particularly in combination with approved antifibrotic agents, on clinical outcomes in PF.

Similarly, elevated ACE has been observed in the bronchoalveolar fluid of patients with fibrotic lung diseases (51). Single-nucleotide polymorphism insertion/deletion (I/D) mutations in the ACE gene can alter its function and activity. These mutations can lead to an increase in ACE activity, contributing to pulmonary inflammation and promoting lung fibrosis. Furthermore, an I/D polymorphism of ACE is linked to COPD (52, 53). A higher frequency of the D allele of the ACE gene is observed in patients with PF compared to healthy controls (54). The ACE I/D gene polymorphism is associated with the elevated risk of PF, particularly in the Chinese Han population (55). Moreover, ACE inhibitors such as captopril demonstrated efficacy in reducing collagen deposition in animal models exposed to irradiation (56–58). The above findings suggest that ACE plays a significant role in promoting the development of PF. These observations indicate that inhibiting Ang II or ACE may serve as a potential therapeutic approach for PF.

## 3 The significance of the ACE2/Ang (1–7)/Mas network in PF

ACE2, the primary receptor for SARS-CoV-2 entry into host cells (59, 60), is widely expressed in various organs, including the lungs (particularly on the surface of alveolar epithelial cells) (61), cardiovascular system, intestine (62), kidneys (63), central nervous system (64), and adipose tissue (65). It is also present in the testes and prostate tissues (66).

The risk of developing PF increases with decreased ACE2 levels in SARS-CoV-2-infected individuals, as ACE2 exerts anti-fibrotic effects post-infection (67, 68). However, reduced ACE2 levels may also offer protection against SARS-CoV-2 infection in susceptible populations, as ACE2 provides binding opportunities for the virus before infection (60, 62). These findings suggest that ACE2 plays distinct roles at different stages of infection.

ACE2 plays a significant role in the RAS by degrading Ang II to generate Ang (1–7) (68, 69). Ang (1–7) mitigates organ fibrosis, including that of the liver and lungs, by binding to MasR, which is encoded by the proto-oncogene Mas (70–72). It also inhibits tumor cell proliferation and modulates inflammation and angiogenesis in various types of tumors (73).

ACE2 and Ang (1–7) levels are significantly reduced following BLM administration (74). Both mRNA expression and activity of ACE2 are significantly decreased in experimental models of lung fibrosis and in patients with PF (75). Previous studies have shown that the alternative RAS pathway, including ACE2, mitigates inflammatory lung disease (76). The ACE2/Ang (1–7)/Mas pathway counteracts the adverse effects of the classical RAS, playing a critical role in regulating physiological and pathological functions in humans. ACE2 can suppress TGF-β1 signaling to inhibit EMT in alveolar epithelial cells induced by lipopolysaccharide (78). Activation of ACE2 using DIZE significantly increases E-cadherin levels while reducing α-SMA, collagen I, vimentin, hydroxyproline, and TGF-β1, therefore mitigating silica-induced lung fibrosis (77). It also modulates the TGF-β1/Smad2/Smad3 signaling pathway in type II alveolar epithelial cells, inhibiting collagen accumulation and TGF-β1 pathway activation (78). Exogenous ACE2 has been shown to attenuate BLM-induced fibrosis by preserving local ACE2 levels and preventing the increase of AGT (80). Overexpression of ACE2/Ang (1–7) reverses increased mRNA levels of TGF-β and other pro-inflammatory cytokines in BLM-treated rat models (79). Furthermore, ACE2 reduces apoptosis in alveolar type II epithelial cells induced by silica (80) while upregulation of ACE2 ameliorates fibrosis and EMT in these cells (81). Inhibiting ACE2, blocking the MasR, or knocking down the ACE2 gene worsens EMT, ECM accumulation, and lung dysfunction in silica-treated mice (83). ACE2-deficient mice show impaired exercise capacity, compromised lung function, and increased collagen deposition following BLM treatment compared to wild-type mice (82). These findings underscore that ACE2 alleviates EMT, ECM deposition, and TGF-β1 levels *in vitro* and *in vivo*, demonstrating its potential as a therapeutic target in lung fibrosis.

Furthermore, suppressing the ACE/Ang II/AT_1_R pathway using acetyl-seryl-asparyl-lysyl-proline, an anti-fibrotic peptide, reduces EMT and abnormal ECM deposition in silica-induced pulmonary interstitial fibrosis. This effect is mediated through the ACE2/Ang (1–7)/Mas pathway stimulation, thus protecting against fibrosis (83). Similarly, Ang (1–7) alleviates EMT induced by TGF-β1 (84). Exogenous Ang (1–7) enhances E-cadherin synthesis, reduces ECM formation induced by TGF-β1, and inhibits the phosphorylation of Smad2 and Smad3 (84). Overexpression of Ang (1–7) similarly decreases the deposition of excessive collagen, reduces mRNA levels of TGF-β, and suppresses the release of pro-inflammatory cytokines (79, 85, 86). Ang (1–7) alleviates EMT and decreases the production of AT1R and Ang II by inhibiting SRC kinase in early PF models induced by lipopolysaccharide. These effects are blocked by the MasR antagonist A779 (87). Collectively, these findings highlight the ACE2/Ang (1–7) pathway as a potent anti-fibrotic, anti-inflammatory, and anti-apoptotic pathway, making it a promising therapeutic target for PF.

## 4 Regulation of autophagy, oxidative stress, and inflammation in PF by classical RAS and ACE2/Ang (1–7)/Mas pathways

**Figure 2:**
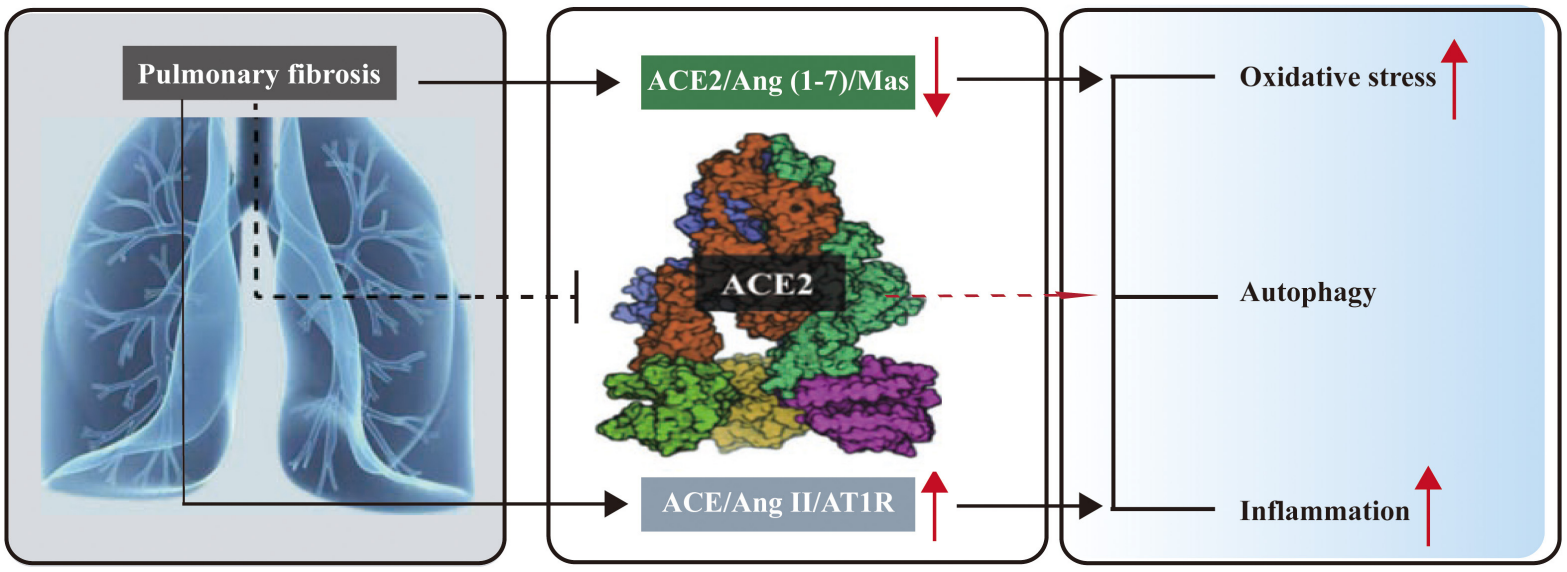
Role of RAS and the ACE2/Ang (1–7)/Mas focuses on ACE2 in pulmonary fibrosis. In pulmonary fibrosis, upregulated ACE/Ang II/AT1R axis and downregulated ACE2/Ang (1–7)/Mas axis exist, which can induce oxidative stress and inflammation. Over-expression of ACE2 can reduce oxidative stress and inflammation.

Increasing evidence suggests that oxidative stress and cytokine production are closely linked to the development of PF (88). Several studies have indicated that over-activated reactive oxygen species (ROS) contribute to the progression of PF (89, 90). Chronic inflammation in fibrosis persists, triggering excessive ROS production and TGF-β synthesis, which leads to fibroblast activation and ECM accumulation. Recent findings have highlighted that the Ang II and ACE2/Ang (1–7)/Mas network plays a significant role in mediating oxidative stress (91–93), autophagy (91, 94), and inflammation (95) during PF (Figure 2).

Ang II induces inflammation and oxidative stress through its interaction with AT_1_R (11, 47, 69). Ang II activates autophagy flux, intercellular ROS production, collagen synthesis, and NOD-like receptor family pyrin domain-containing 3 (NLRP3) expression. The profibrotic effect of BLM was reversed by autophagy inhibitors such as rapamycin and 3-MA, suggesting that inhibiting autophagy has an antifibrotic role in PF (91). The imbalance of autophagy caused by oxidative stress leads to increased ROS and apoptosis. ROS levels and oxidative stress markers are also upregulated in patients with PF, and high ROS levels are associated with poor prognosis. Combining pirfenidone and losartan (an AT_1_R antagonist) may provide stronger protection against PF than monotherapy by enhancing anti-inflammatory and antioxidant effects (96). A previous study suggested that ACE2 may regulate autophagy, as the autophagy inhibitor 3-MA mitigated the severity of ALI induced by lipopolysaccharide (LPS) (97). Furthermore, Ang (1–7) reduced NADPH oxidase 4 (NOX4) protein levels and inhibited autophagy, improving PF induced by smoking (98). Inhibiting autophagy also improved lung fibrosis in BLM-treated animals (91), which could be attributed to differences in the experimental models. However, contradictory reports exist regarding autophagy regulation by Ang II and the ACE2/Ang (1–7)/Mas network in PF. Overexpression of ACE2 in mice treated with BLM resulted in less collagen deposition and lower levels of NOX4, but higher LC3-II protein levels, indicating that ACE2 overexpression alleviated PF by enhancing autophagy (94). This suggests that autophagy may exert a dual role in PF. The seemingly contradictory reports regarding the role of ACE2-mediated autophagy in PF underscore the complexity of its dynamic regulatory networks and intricate microenvironmental influences during disease progression. To elucidate the precise mechanisms of the ACE2–autophagy axis across diverse etiologies and disease stages, future investigations should integrate cutting-edge single-cell sequencing technologies, dynamic pathological modeling, and comprehensive clinical cohort analyses.

The combination of AT_1_R antagonist losartan with pirfenidone reduced the release of inflammatory factors, such as interleukin-1β, tumor necrosis factor-α, TGF-β1, and platelet-derived growth factor, and reduced collagen formation. This suggests that the combined therapy has anti-inflammatory and anti-fibrotic effects in PF models treated with BLM (99). Overexpression of ACE2 in umbilical cord mesenchymal stem cells (ACE2-UCMSCs) has been shown to be more effective in reducing collagen deposition than either ACE2 or UCMSCs alone. In the ACE2-UCMSCs treatment group, fibrosis severity was attenuated, accompanied by a reduction in the release of inflammatory cytokines, including IL-1, IL-2, IL-6, and IL-10. These findings suggest that ACE2 and UCMSCs exert a synergistic effect on lung fibrosis caused by BLM (100). Bone marrow-derived mesenchymal stem cells (MSCs) overexpressing ACE2 improved the release of inflammatory mediators and pulmonary endothelial function in ALI induced by lipopolysaccharide (101). Exogenous Ang (1–7) and ACE2 together can reduce the synthesis and release of cytokines and chemokines, inhibit the migration of inflammatory cells to the lung, and improve pulmonary function (102, 103). Ang (1–7) significantly suppresses NADPH oxidase activation and inhibits nitric oxide synthase (NOS) release induced by both Ang II and IL-1β. Ang (1–7) can alleviate Ang II-driven vascular smooth muscle cell inflammation (104). Downstream cascades of Ang (1–7) help mitigate inflammation and fibrosis through the MasR (71, 95). Some studies have suggested that the anti-fibrotic effects of ALA (alpha-lipoic acid) occur by blocking oxidative stress and promoting autophagy. ALA also reduced the deposition of ECM components (such as collagen I and α-SMA) in fibroblasts challenged by Ang II, and its effects were suppressed by D-Pro7-Ang (1–7), a MrgD antagonist. These findings indicate that ALA alleviates PF by suppressing oxidative stress and activating autophagy (105, 106).

In human endothelial cells, Ang (1–7) enhances the release of nitric oxide (NO) and prostaglandins, promoting vasodilation by counteracting the vasoconstrictor effects of Ang II mediated by AT_1_R (107). The absence of NO exacerbates fibrotic changes in PF mice induced by BLM (108). NO also inhibits the release of connective tissue growth factor by blocking the Smad-dependent TGF-β signaling pathway. In cellular models, exogenous Ang (1–7) and ACE2 reduced inflammation and accumulation of collagen I induced by Ang II by inhibiting the MAPK/NF-κB pathway. These effects were reversed by the Mas inhibitor, A-779 (109). However, continuous infusion of Ang (1–7) paradoxically exacerbates lung inflammation. This paradox could be explained by the fact that, when the ACE/Ang II/AT_1_R pathway is stimulated by BLM or Ang II, exogenous Ang (1–7) suppresses the protein expressions of ACE/Ang II/AT_1_R while promoting the expression of ACE2, Ang (1–7), and Mas, activating Mas (an antagonist of AT_1_R) and inhibiting Ang II. However, Ang (1–7) may play a pro-inflammatory role when binding to AT_1_R in the absence of ACE/Ang II/AT_1_R stimulation (109). Furthermore, increased NO mediated by AT_2_R has been shown to reduce the production of pro-inflammatory cytokines and enhance the production of anti-inflammatory cytokines (110). Blocking MasR with A779 prevented the deposition of ECM. The Ang (1–7)/MasR pathway is also involved in the anti-inflammatory and anti-fibrotic effects of aerobic training in chronic asthma models (111). These findings suggest that the ACE2/Ang (1–7)/Mas pathway can reduce inflammation in lung fibrosis by increasing NO production and suppressing the expression of inflammatory factors.

## 5 Conclusion and perspectives

The over-activation of the ACE/Ang II/AT_1_R network results in an imbalance between the classic RAS and the ACE2/Ang (1–7)/Mas pathway, contributing to the initiation and progression of PF. ACE2, as an inverse modulator of the local RAS, facilitates the formation of Ang (1–7) from Ang II, thus regulating local Ang II levels and counteracting its harmful effects. Pharmacological agents that target the ACE/Ang II/AT_1_R network and upregulate the ACE2/Ang (1–7)/Mas pathway could offer promising therapeutic strategies for the treatment of PF in the future.
